# N-Cadherin Regulates the Odontogenic Differentiation of Dental Pulp Stem Cells via β-Catenin Activity

**DOI:** 10.3389/fcell.2021.661116

**Published:** 2021-03-30

**Authors:** Zilong Deng, Wenjuan Yan, Xingzhu Dai, Ming Chen, Qian Qu, Buling Wu, Wanghong Zhao

**Affiliations:** ^1^Department of Stomatology, Nanfang Hospital, Southern Medical University, Guangzhou, China; ^2^Stomatological Hospital, Southern Medical University, Guangzhou, China; ^3^Stomatology Healthcare Center, Shenzhen Maternity and Child Healthcare Hospital, Shenzhen, China; ^4^Shenzhen Stomatology Hospital (Pingshan), Southern Medical University, Shenzhen, China

**Keywords:** dental pulp stem cells, pulp regeneration, odontogenic differentiation, N-cadherin, β-catenin

## Abstract

Dental pulp stem cell (DPSC) transplantation has shown new prospects in dental pulp regeneration, and is of great significance in the treatment of pulpitis and pulp necrosis. The fate and regenerative potential of stem cells are dependent, to a great extent, on their microenvironment, which is composed of various tissue components, cell populations, and soluble factors. N-cadherin-mediated cell–cell interaction has been implicated as an important factor in controlling the cell-fate commitment of mesenchymal stem cells. In this study, the effect of N-cadherin on odontogenic differentiation of DPSCs and the potential underlying mechanisms, both *in vitro* and *in vivo*, was investigated using a cell culture model and a subcutaneous transplantation mouse model. It was found that the expression of N-cadherin was reversely related to the expression of odontogenic markers (dentin sialophosphoprotein, DSPP, and runt-related transcription factor 2, Runx2) during the differentiation process of DPSCs. Specific shRNA-mediated knockdown of N-cadherin expression in DPSCs significantly increased the expression of DSPP and Runx2, alkaline phosphatase (ALP) activity, and the formation of mineralized nodules. Notably, N-cadherin silencing promoted nucleus translocation and accumulation of β-catenin. Inhibition of β-catenin by a specific inhibitor XAV939, reversed the facilitating effects of N-cadherin downregulation on odontogenic differentiation of DPSCs. In addition, knockdown of N-cadherin promoted the formation of odontoblast-like cells and collagenous matrix in β-tricalcium phosphate/DPSCs composites transplanted into mice. In conclusion, N-cadherin acted as a negative regulator via regulating β-catenin activity during odontogenic differentiation of DPSCs. These data may help to guide DPSC behavior by tuning the N-cadherin-mediated cell–cell interactions, with implications for pulp regeneration.

## Introduction

Dental caries is one of the most common bacterial infectious diseases worldwide, leading to destruction of the tooth structure and irreversible pulpitis or pulp necrosis. Root canal therapy is the common endodontic treatment for pulp diseases that involves the extirpation of diseased pulp and the subsequent obturation of root canal systems with bioinert synthetic materials, resulting in a permanently inactivated tooth that is more prone to fractures and re-infection (Gong et al., 2016). In recent years, the advancement of tissue engineering and regenerative medicine have greatly spurred the development of regenerative endodontics, which seeks to replace the inflammatory/necrotic pulp tissue with regenerated pulp-like tissue to reestablish the protective functions, including sensibility, healing, innate and adaptive immunity (Cao et al., 2015). Remarkably, dental pulp stem cell (DPSC)-mediated approaches for regeneration have demonstrated promising results in terms of generating dental pulp-like tissues with morphologic characteristics that resemble those of normal dental pulp *in vivo* and *in situ* (Gronthos et al., 2000, 2002; Huang et al., 2010; Nakashima et al., 2017; Xuan and Li, 2018). Nevertheless, the effectiveness of stem cell-based regeneration is not always realized, because of the dynamic and complex microenvironment in which it is applied. The fates and regenerative potential of stem cells are dependent on their microenvironment, which is composed of various tissue components, cell populations, and soluble factors (Zheng et al., 2019; Sui et al., 2020). Therefore, the improvement of regenerative endodontic procedures requires a better understanding of how the microenvironment controls DPSC behavior.

Cell–cell and cell–extracellular matrix (ECM) interactions mediated by adhesion molecules (cadherins and integrins, respectively) are important mechanisms controlling tissue morphogenesis and cell fate (Marie et al., 2014b). Previously, we found that knockdown of Integrin α5 in DPSCs impairs proliferation and migration, while enhancing their odontogenic differentiation capacity (Cui et al., 2014; Xu et al., 2015). Further, the Integrin α5 priming synthetic cyclic peptide promotes the deposition of ECM, the activity of ECM-receptor, and hence the odontogenic differentiation of DPSCs (Wang et al., 2019).

However, how cadherins regulate odontogenic differentiation of DPSCs remains largely unknown. Cadherins are composed of an extracellular domain that mediates calcium-dependent homophilic interactions between cadherin molecules, a transmembrane domain, and an intracellular domain (Derycke and Bracke, 2004). The intracellular domain interact with cytoskeletal proteins, allowing cell anchorage, and with several signaling molecules, including vinculin, α-catenin, and β-catenin, indicating the involvement of cadherins in modulating cellular signaling processes in addition to cell–cell adhesion (Marie et al., 2014b). In mesenchymal cells, cell–cell interaction is mediated through N-cadherin (Cosgrove et al., 2016). Thus, in this study, the effect of N-cadherins on the odontogenic differentiation of DPSCs, and the potential underlying mechanism, was investigated. Further, the *in vivo* effect was examined using a subcutaneous transplantation mouse model.

## Materials and Methods

### Cell Culture and Treatment

Healthy dental pulp tissues were collected from caries-free teeth of patients (age 18–25 years old) undergoing extraction of fully erupted third molars, according to the informed protocol approved by the Ethics Committee of Nanfang Hospital, Southern Medical University, Guangzhou, China. Primary human DPSCs were isolated, identified and cultured in Dulbecco’s modified Eagle’s medium (Gibco, Grand Island, NY, United States) supplemented with 10% fetal bovine serum (Gibco) and 1% penicillin/streptomycin (Gibco), as described previously (Chen et al., 2020). The cells were maintained at 37°C in a humidified atmosphere with 5% CO_2_ and 95% air. Cells cultured for 3–5 passages were used for the following experiments.

To induce odontogenic differentiation, the cells were incubated in odontogenic medium consisting of basal medium, 10 nM dexamethasone (Sigma-Aldrich, St. Louis, MO, United States), 50 μg/mL ascorbic acid 2-phosphate (Sigma-Aldrich), and 10 mM β-glycerophosphate (Sigma-Aldrich) for the indicated times. The medium was changed every 2–3 days. For the β-catenin pathway inhibition experiment, cells were cultured with 2 μM XAV939 (Selleck, Houston, TX, United States). Dimethyl sulfoxide (DMSO) (<0.2%) was used as for the vehicle-only control.

### Quantitative Real-Time Polymerase Chain Reaction

Total RNA was extracted from DPSCs using the TRIzol reagent (Invitrogen, Carlsbad, CA, United States) following the manufacturer’s instructions. Complementary DNA synthesis was performed using the PrimeScript^TM^ RT Master Mix (TaKaRa, Kyoto, Japan). Quantitative PCR analyses were performed using the LightCycler 480 Real-time PCR System (Roche, Indianapolis, IN, United States) with SYBR^®^ Premix Ex Taq^TM^ (TaKaRa). The 2^–Δ^ ^Δ^ ^*Ct*^ value was used to calculate the relative fold change of gene expression normalized to an internal control (β-actin). The primer sequences used were as follows:

N-cadherin: forward 5′-TCTGGGTCTGTTTTATTACTCCTGG-3′, reverse 5′-GCGAGCTGATGACAAATAGCG-3′;

Runx2: forward 5′-CACTGGCGCTGCAACAAGA-3′, reverse 5′-CATTCCGGAGCTCAGCAGAATAA-3′;

DSPP: forward 5′-TCACAAGGGAGAAGGGAATG-3′, reverse 5′-TGCCATTTGCTGTGATGTTT-3′;

β-actin: forward 5′-CCATCGTCCACCGCAAAT-3′, reverse 5′-CCTGTAACAACGCATCTCATA-3′.

### Western Blotting

Total protein was extracted from DPSCs using RIPA buffer in the presence of protease inhibitor and phosphatase inhibitor (Beyotime, Shanghai, China) following the manufacturer’s instructions. The Nuclear and Cytoplasmic Protein Extraction Kit (Beyotime) was used in cases where the extraction of separate cytoplasmic and nuclear protein fractions was necessary. The Enhanced BCA Protein Assay Kit (Beyotime) was used to measure the protein concentrations. Proteins were separated by sodium dodecyl sulfate-polyacrylamide gel electrophoresis, transferred to polyvinylidene fluoride membranes (EMD Millipore, Billerica, MA, United States), and then incubated with primary antibodies overnight at 4°C, followed by horseradish peroxidase-conjugated secondary antibodies for 1 h at room temperature. The following primary antibodies were used: anti-N-cadherin (sc-59987, 1:1000; Santa Cruz, Dallas, TX, United States), anti-Runx2 (sc-390715, 1:1000; Santa Cruz), anti-DSPP (sc-73632, 1:1000; Santa Cruz), anti-β-catenin (ab32572, 1:5000; Abcam, Cambridge, United Kingdom), anti-Lamin B1 (ab16048, 1:1000; Abcam), and anti-β-actin (ab8226, 1:5000; Abcam). Proteins were visualized using enhanced chemiluminescence substrate. Protein levels were normalized to the β-actin or Lamin B1 signal.

### Lentivirus Transfection

A lentivirus expressing a short hairpin RNA (shRNA) specific to the N-cadherin (oligo sequence: CCGGCCTAAG ATCATTCGCCAAGAACTCGAGTTCTTGGCGAATGATCTT AGGTTTTTG) and a negative control lentivirus (oligo sequence: CCGGTTCTCCGAACGTGTCACGTCTCGAGACGTGACACG TTCGGAGAATTTTTG) were provided by GeneChem (Shanghai, China). DPSCs were seeded into 6-well plates at a density of 6 × 10^4^ cells/well and were infected with viral supernatants (multiplicity of infection = 50) supplemented with polybrene (5 μg/mL) for 10 h, and then incubated under normal growth conditions for a further 72 h. After screening with puromycin (1 μg/mL), DPSCs stably expressing the N-cadherin-specific shRNA were established. The knockdown efficiency was confirmed by quantitative real-time polymerase chain reaction (qPCR) and western blotting.

### Alkaline Phosphatase Staining and Activity

After culture in odontogenic medium for 7 days, DPSCs were fixed with 4% paraformaldehyde for 30 min at room temperature and washed with distilled water. Alkaline phosphatase (ALP) staining was performed with the BCIP/NBT ALP Color Development Kit (Beyotime) according to the manufacturer’s instructions. The total protein content was determined in the same sample by an ALP activity kit (Jiancheng, Nanjing, China). The absorbance of each well was determined by measurements at 520 nm. ALP activity relative to the control treatment was calculated after normalization to the total protein content.

### Alizarin Red S Staining and Quantification

After culture in odontogenic medium for 14 days, DPSCs were fixed with 4% paraformaldehyde for 30 min at room temperature and washed with distilled water. Calcium deposition in the ECM was stained with 2% Alizarin Red S (ARS) (pH 4.2; Sigma-Aldrich) for 15 min. The mineralized nodules were observed and photographed using an inversed microscope (Olympus, Tokyo, Japan). To further quantify the mineralized nodules, the stain was dissolved in 10% cetylpyridinium chloride (Sigma-Aldrich) for 1 h, and the calcium concentration was determined by absorbance measurements at 562 nm.

### Immunofluorescence Staining

Dental pulp stem cells cultured on coverslips were fixed with 4% paraformaldehyde at 4°C for 15 min, and then permeabilized with 0.3% Triton X-100 for 5 min. After blocking with 10% normal goat serum for 1 h, the cells were incubated at 4°C overnight with anti-β-catenin (ab32572, 1:250; Abcam) primary antibody. Subsequently, the cells were incubated at 37°C for 1 h in the dark with goat anti-rabbit conjugated with Alexa Fluor 647 (ab150079, 1:1000; Abcam) secondary antibody. 4′,6-diamidino-2-phenylindole (DAPI) (Roche) was applied to visualize the nuclei. Images were captured using a fluorescent microscope (Olympus).

### DPSC Subcutaneous Transplantation

Animal experiments were performed in accordance with the regulations of the Ethics Committee of Nanfang Hospital, Southern Medical University, Guangzhou, China. Five male BALB/c immunocompromised nude mice (weight: 15–18 g; age: 6 weeks) were purchased from the Medical Laboratory Animal Center of Guangdong Province (China) and were maintained under pathogen-free conditions in the Experimental Animal Center at Nanfang Hospital, Southern Medical University.

Beta-tricalcium phosphate (β-TCP) blocks were provided by the Biological Materials Manufacturing Core, Sichuan University. DPSCs were transfected with either the control or N-cadherin-specific shRNA lentivirus, and a total of 1 × 10^6^ cells were loaded onto the β-TCP scaffold per block. The composites of β-TCP blocks and cells were transplanted into the left and right dorsal subcutaneous region of mice as previously described (Li et al., 2018). Four weeks after transplantation, the mice were euthanized, and the composites were harvested, fixed in 4% paraformaldehyde at 4°C for 48 h, decalcified in 10% EDTA (pH 7.4) at room temperature for 10 days, and embedded in paraffin.

### Hematoxylin and Eosin Staining and Masson’s Trichrome Staining

The embedded samples were serially sectioned at 4-μm thickness. The sections were deparaffinized in xylene, rehydrated through a series of graded ethanol solutions, and stained with hematoxylin and eosin (H&E) or Masson’s trichrome (Solarbio, Beijing, China) according to the manufacturer’s instructions.

### Immunohistochemistry Staining

The sections were dehydrated, subjected to antigen retrieval, and incubated with primary antibodies at 4°C overnight, followed by a horseradish peroxidase-conjugated secondary antibody for 40 min at room temperature. The immunostained proteins were visualized by the application of diaminobenzidine solution. The following primary antibodies were used: anti-DSPP (sc-73632, 1:500; Santa Cruz), anti-nestin (ab105389, 1:100; Abcam), and anti-β-catenin (ab32572, 1:500; Abcam). The sections were lightly counterstained with hematoxylin, mounted, and analyzed under a light microscope (Olympus) by a technician who was blinded to the samples. The number of positive cells exhibiting brown staining were counted at 400× the original magnification in five randomly picked fields per slide, and the mean value of these fields was calculated for each group.

### Statistical Analyses

All experiments were repeated at least thrice. Data are presented as the means ± SEM unless otherwise stated and were analyzed using SPSS software (version 21.0; IBM, Chicago, IL, United States). Data normality and homogeneity of variances were confirmed with Shapiro–Wilk test and *F*-test, respectively. Samples with normal distribution were analyzed with the independent sample *t*-test for two groups or one-way analysis of variance (ANOVA) followed by Bonferroni post-test for multiple groups. Samples without normal distribution were analyzed using the non-parametric Mann–Whitney *U* test for two groups or Kruskal–Wallis test for multiple groups. The statistical significance level was set at *P* < 0.05.

## Results

### The Expression of N-Cadherin Was Decreased During Odontogenic Differentiation of DPSCs

**FIGURE 1:**
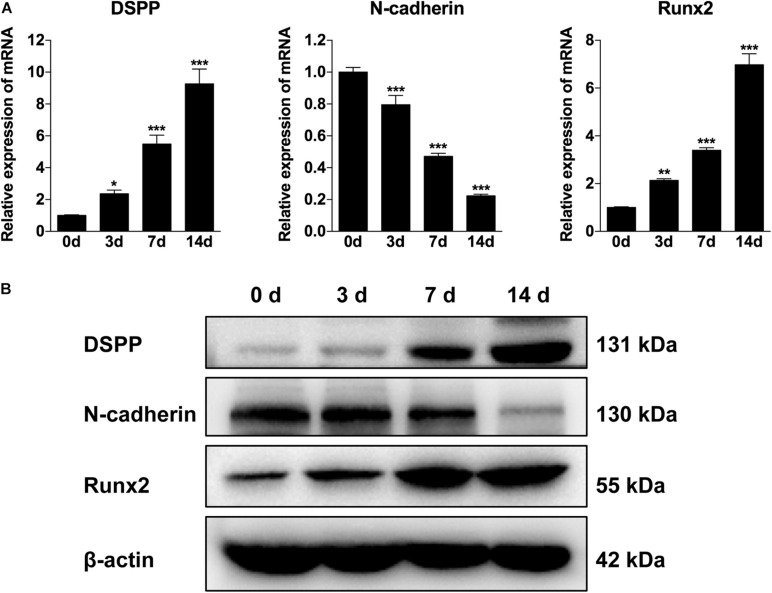
The expression of N-cadherin was decreased during odontogenic differentiation of dental pulp stem cells (DPSCs). DPSCs were cultured in odontogenic medium for 3, 7, and 14 days, and then the expression pattern of N-cadherin and odontogenic markers (dentin sialophosphoprotein, DSPP, and runt-related transcription factor 2, Runx2) were examined by qPCR **(A)** and western blot **(B)** analysis. Data are presented as the mean ± SEM of three independent experiments. *P < 0.05, **P < 0.01, ***P < 0.001.

To explore the involvement of N-cadherin in the odontogenic differentiation of DPSCs, DPSCs were cultured in odontogenic medium for 3, 7, and 14 days, and then the expression pattern of N-cadherin and odontogenic markers (DSPP and Runx2) were examined by qPCR and western blot analysis. The expression of DSPP and Runx2 gradually increased over time as expected, whereas the expression of N-cadherin gradually decreased both at the mRNA and protein level (Figures 1A,B). These results indicate that a functional decrease in N-cadherin was required during odontogenic differentiation of DPSCs.

### N-Cadherin Knockdown Promoted Odontogenic Differentiation of DPSCs

**FIGURE 2:**
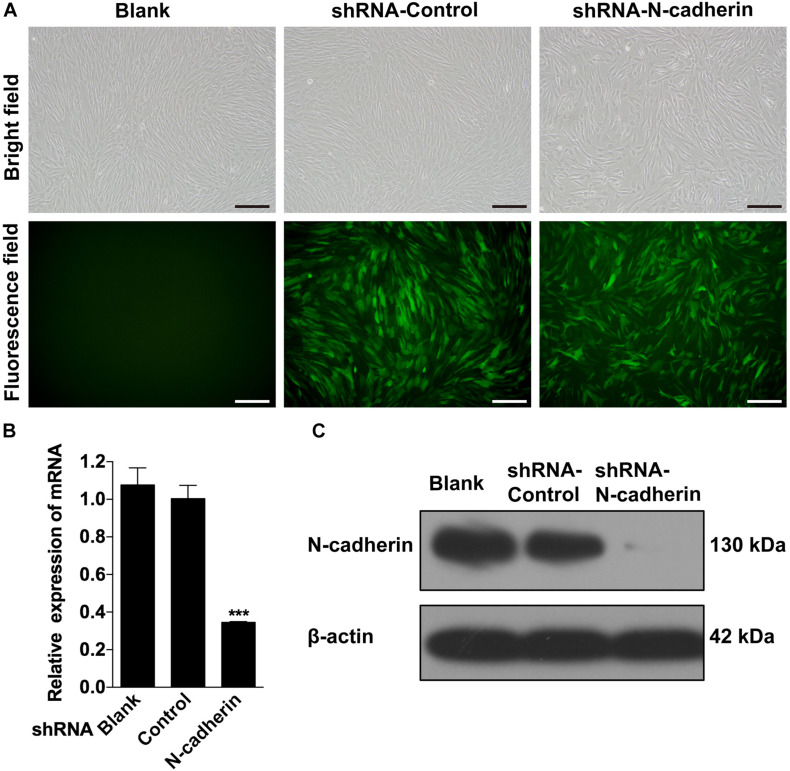
Stable downregulation of N-cadherin in dental pulp stem cells (DPSCs). DPSCs were transfected with either the N-cadherin shRNA or control shRNA lentivirus. The transfection efficiency was evaluated using fluorescence microscopy **(A)**. The expression level of N-cadherin mRNA and protein were detected by qPCR and western blotting, respectively **(B,C)**. Scale bars = 200 μm. Data are presented as the mean ± SEM of three independent experiments. ***P < 0.001.

Given that there is a negative correlation between N-cadherin and the odontogenic markers, it was investigated whether inhibition of N-cadherin could promote DPSC differentiation by knocking-down N-cadherin via lentiviral transfection (Figure 2A). qPCR and western blot analysis showed that N-cadherin was significantly downregulated both at the mRNA and protein level after N-cadherin shRNA lentivirus transfection in DPSCs (Figures 2B,C).

**FIGURE 3:**
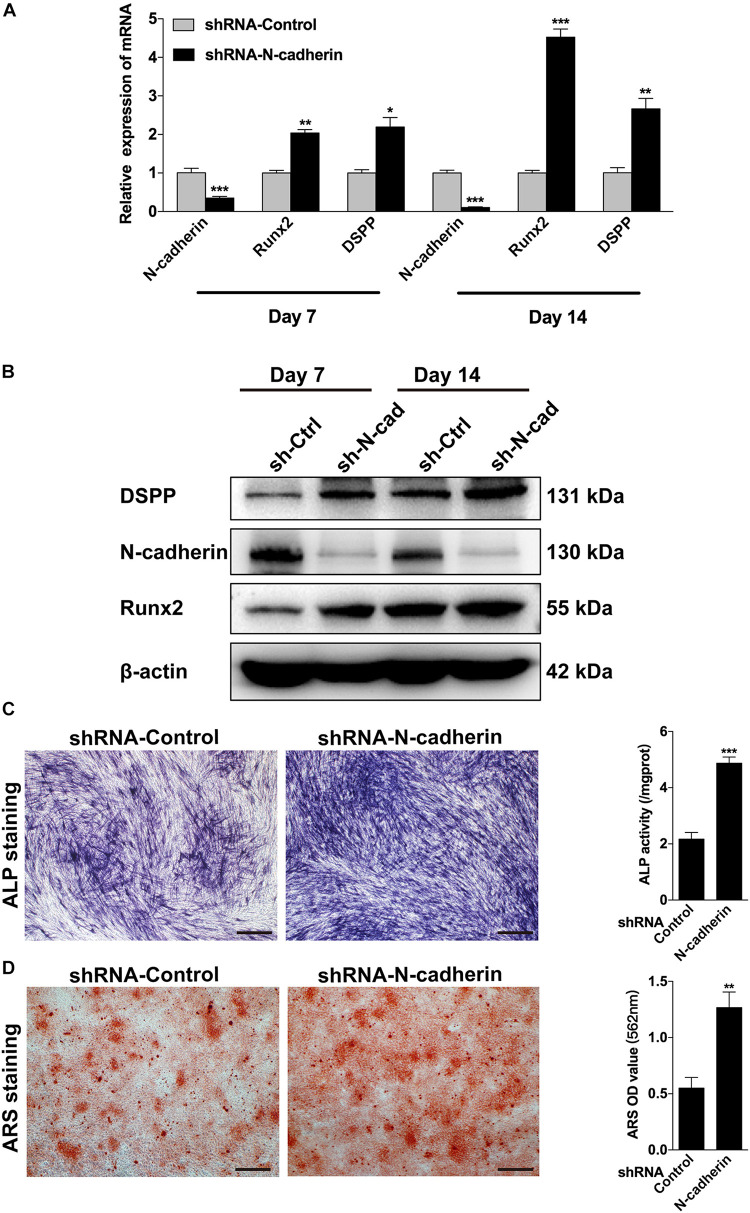
N-cadherin knockdown promoted the odontogenic differentiation of dental pulp stem cells (DPSCs). DPSCs transfected with a control or N-cadherin-specific shRNA lentivirus were subjected to odontogenic induction for 7 or 14 days. The expression level of odontogenic-related genes (DSPP and Runx2) were detected by qPCR and western blotting on day 7 and 14 **(A,B)**. The alkaline phosphatase (ALP) activity was measured by ALP staining on day 7 **(C)**. The formation of mineralized nodules was detected by alizarin red staining (ARS) on day 14 **(D)**. Scale bars = 500 μm. Data are presented as the mean ± SEM of three independent experiments. *P < 0.05, **P < 0.01, ***P < 0.001.

Next, lentivirus-transfected DPSCs were subjected to odontogenic differentiation. Following induction for 7 and 14 days, ALP staining, alizarin red staining, qPCR, and western blotting were performed to identify the differentiation of DPSCs. Compared with the control shRNA lentivirus-transduced cells, the expression of odontogenic-related genes, DSPP and Runx2, were significantly upregulated in the N-cadherin shRNA lentivirus-transduced cells (Figures 3A,B). Meanwhile, ALP activity and the presence of mineralized nodules were increased in N-cadherin shRNA lentivirus-transduced cells (Figures 3C,D). These results suggest that N-cadherin acted as a negative regulator during odontogenic differentiation of DPSCs.

### N-Cadherin Downregulation Enhanced the Odontogenic Differentiation of DPSCs via Increasing β-Catenin Activity

**FIGURE 4:**
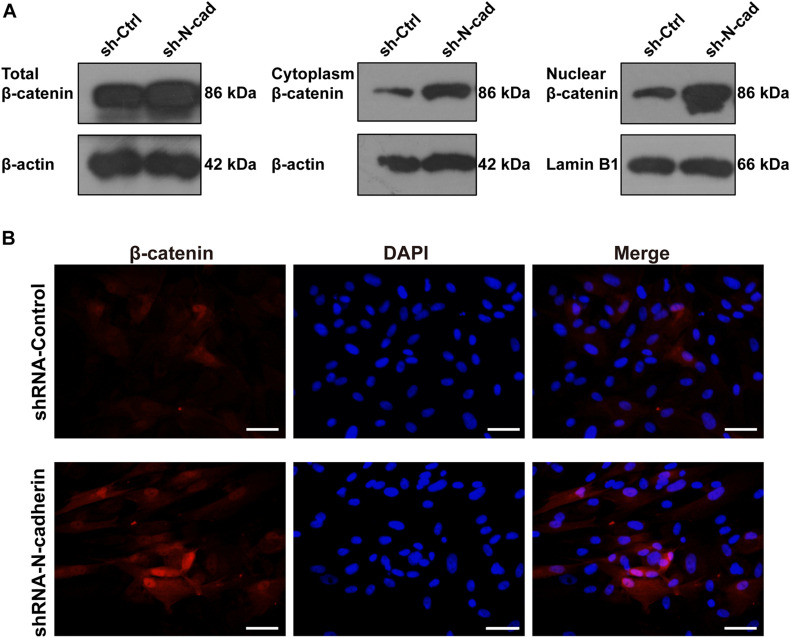
N-cadherin downregulation enhanced β-catenin activity in dental pulp stem cells (DPSCs). DPSCs transfected with a control or N-cadherin-specific shRNA lentivirus were subjected to odontogenic induction for 14 days. The expression level of nuclear, cytoplasmic, and total β-catenin was detected by western blotting **(A)**. The nuclear translocation of β-catenin was detected by immunofluorescent staining. Nuclei were counterstained with DAPI **(B)**. Scale bars = 50 μm.

β-catenin is involved in both cadherin-mediated cell adhesion and the canonical Wnt/β-catenin pathway. To determine nucleus translocation and accumulation of β-catenin, a marker for β-catenin signaling activation was detected by western blotting and immunofluorescent staining. As shown in Figure 4A, shRNA-mediated knockdown of N-cadherin increased the expression of β-catenin in the nucleus and cytoplasm, as well as the expression of total β-catenin. In line with this, silencing of N-cadherin promoted the translocation of β-catenin to the nucleus (Figure 4B). These results indicate that the inhibition of N-cadherin increased β-catenin activity during the odontogenic differentiation of DPSCs.

**FIGURE 5:**
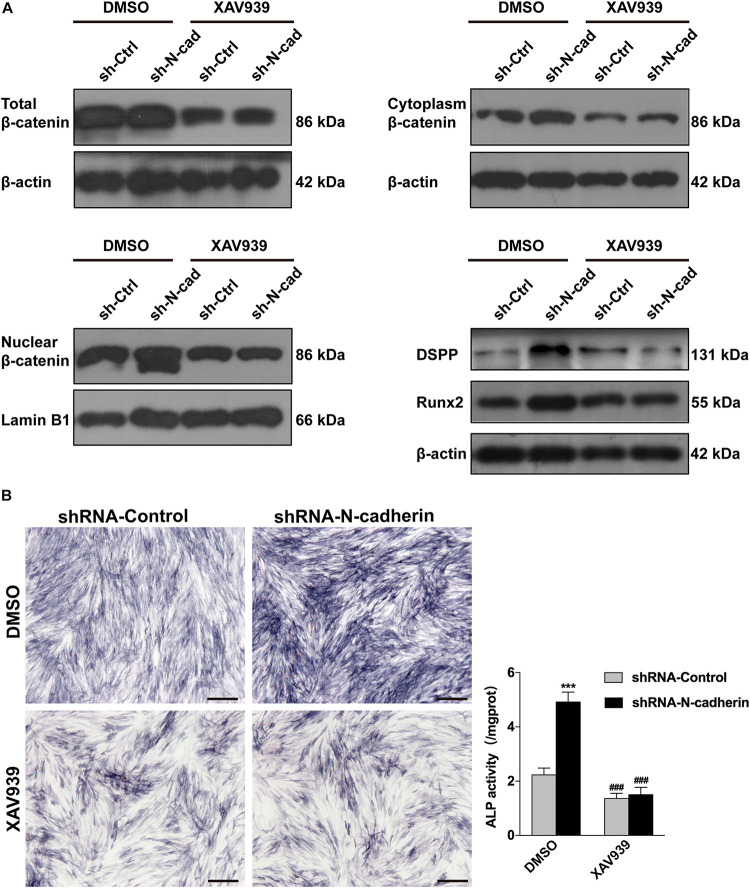
Inhibition of β-catenin by XAV939 reversed the effects of N-cadherin downregulation on dental pulp stem cells (DPSCs) odontogenic differentiation. DPSCs transfected with a control or N-cadherin-specific shRNA lentivirus were subjected to odontogenic induction in the presence or absence of XAV939 (a β-catenin inhibitor). DMSO was used as the vehicle-only control. The expression of nuclear, cytoplasmic, and total β-catenin, and the odontogenic-related genes (DSPP and Runx2), were detected by western blotting on day 14 **(A)**. The alkaline phosphatase (ALP) activity was measured by ALP staining on day 7 **(B)**. Scale bars = 500 μm. Data are presented as the mean ± SEM of three independent experiments. ***P < 0.001 compared with the shRNA-Control group, ^###^*P* < 0.001 compared with the DMSO group.

Further, lentivirus-transfected DPSCs were treated with XAV939, a specific inhibitor of β-catenin. As expected, XAV939 decreased the expression of nuclear, cytoplasmic, and total β-catenin, as well as odontogenic-related genes (DSPP and Runx2) (Figure 5A). Consistently, ALP activity was also decreased by XAV939 (Figure 5B). These results suggest that inhibition of β-catenin by XAV939 could reverse the effects of N-cadherin downregulation on DPSC odontogenic differentiation.

### N-Cadherin Downregulation Enhanced β-Catenin Activity and Odontogenic Differentiation *in vivo*

**FIGURE 6:**
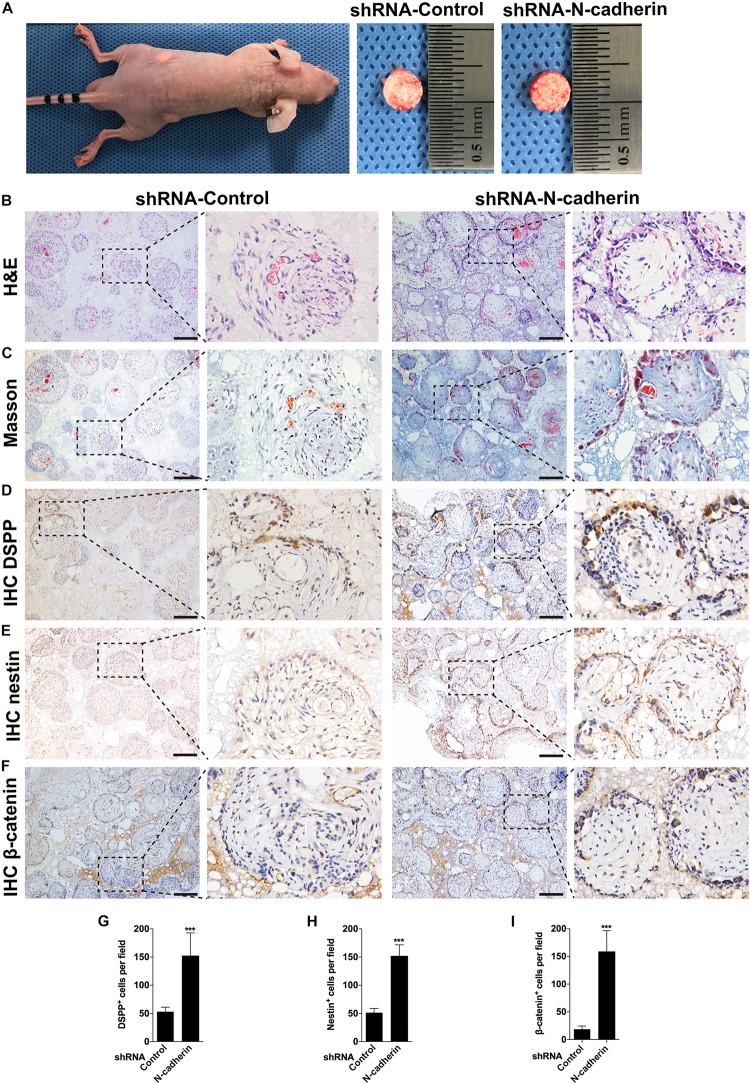
N-cadherin downregulation enhanced β-catenin activity and odontogenic differentiation *in vivo*. Dental pulp stem cells transfected with a control or N-cadherin-specific shRNA lentivirus were loaded onto the β-tricalcium phosphate scaffold, and transplanted subcutaneously into immunocompromised nude mice for 4 weeks **(A)**. The samples were subjected to hematoxylin and eosin (H&E) staining **(B)**, Masson’s trichrome staining **(C)**, DSPP, nestin and β-catenin immunohistochemistry (IHC; **D–F**, respectively). The numbers of DSPP, nestin and β-catenin positive cells were quantified (**G–I**, respectively). Scale bars = 200 μm. Data are presented as the mean ± SD (*n* = 5 per group). ***P < 0.001.

To confirm the impact of N-cadherin on odontogenic differentiation of DPSCs *in vivo*, we performed subcutaneous transplantation with β-TCP/DPSCs composites in BALB/c immunocompromised nude mice for 4 weeks (Figure 6A). H&E staining showed a pulp-like structure, comprised of a thick layer of cells lining along the surfaces of β-TCP scaffold’s macropores and an interstitial tissue infiltrated with blood vessels in the shRNA-N-cadherin group. By contrast, the shRNA-control group showed a disordered structure with cells evenly distributed throughout the whole tissue (Figure 6B). Masson’s trichrome staining revealed an increase of collagenous matrix within the scaffold’s macropores of the shRNA-N-cadherin group (Figure 6C). The layer of cells formed in the shRNA-N-cadherin group were further identified to be odontoblast-like cells with positive DSPP and nestin staining (Figures 6D,E). Similarly, immunohistochemical analysis revealed the presence of β-catenin localized in the nuclei of the cells (Figure 6F). The number of cells that were positive for DSPP, nestin or β-catenin were significantly higher in the shRNA-N-cadherin group than in the shRNA-control group (Figures 6G–I). These results suggest that knockdown of N-cadherin promoted the formation of odontoblast-like cells and collagenous matrix in β-TCP/DPSCs composites transplanted into mice.

## Discussion

The fundamental goal of endodontic treatment is to retain the natural dentition. Regeneration of a functional pulp-dentin complex is promising for the overall prognosis of the tooth (He et al., 2017). To regenerate the pulp-dentin complex, an appropriate microenvironment is of great importance for stimulating the differentiation of DPSCs. The current study aimed to clarify the involvement of N-cadherin, mediator of cell–cell adhesion, in the odontogenic differentiation of DPSCs. We found that a functional decrease of N-cadherin level was required during the odontogenic differentiation of DPSCs. Artificial downregulation of N-cadherin expression in DPSCs significantly upregulated β-catenin signaling, and consequently enhanced the odontogenic differentiation *in vitro*, which was abrogated by pretreatment of the cells with XAV939, a specific inhibitor of β-catenin. In addition, knockdown of N-cadherin promoted the formation of odontoblast-like cells and collagenous matrix in β-TCP/DPSC composites transplanted into mice.

Mounting evidence shows that the mechanical and biochemical signals from cell–cell adhesion are key to stem cell fate decision (Alimperti and Andreadis, 2015). It was reported that during differentiation of odontoblastic MDPC-23 cells, E-cadherin expression gradually increased over time, whereas the expression of N-cadherin gradually decreased (Lee et al., 2014). Similarly, N-cadherin was expressed in undifferentiated dental bud stem cells, and its expression remained constant at the early stage, but decreased at the late stage of osteogenic differentiation (Di Benedetto et al., 2015). Overexpression of N-cadherin in bone marrow-derived mesenchymal stem cells inhibited osteogenesis and ectopic bone formation, while silencing N-cadherin could promote osteogenesis *in vitro* (Xu et al., 2013). In accordance with these findings, this study determined that the expression of N-cadherin was decreased over time and was reversely related to the expression of odontogenic markers during the differentiation process. Furthermore, knockdown of N-cadherin promoted the odontogenic differentiation of DPSCs *in vitro* and *in vivo*. Collectively, a functional decrease of N-cadherin expression appears to be a universal phenomenon during odontogenic/osteogenic differentiation. This may be explained by lessons learned from tooth development, since there are parallels between pulp development and regeneration (Schmalz and Smith, 2014). During early steps of odontogenesis, N-cadherin initiates dental mesenchymal condensation, which is a fundamental mechanism involved in morphogenesis by facilitating cell–cell interactions (Xiao and Tsutsui, 2012; Giffin et al., 2019). However, as mesenchymal development progresses, the ECM gradually deposits and the microenvironment changes from one that rich in cell–cell interactions to one that dominated by cell–ECM interactions (Cosgrove et al., 2016). In this situation, N-cadherin-mediated cell–cell interactions are increasingly restrained and then become less predominant owing to the dense surrounding matrix. Hence, the fine-tuning of cell–cell and cell–matrix interactions in different phases of odontogenesis is crucial for allowing differentiation to proceed. In addition, integrins and cadherins are linked to each other through the intracellular actin–myosin network. And such linkage regulates the intracellular forces, which is a central mechanism underlying cell fate determination (Mui et al., 2016).

It is well-established that the role of N-cadherin involves both cell–cell interactions and interference with intracellular signaling, particularly the Wnt/β-catenin pathway. N-cadherin binds to β-catenin and modulates its cytoplasmic pools and transcriptional activity (Marie et al., 2014a). In addition, N-cadherin also interferes with low-density lipoprotein receptor-related protein 5 and 6 (LRP5/6) signaling by sequestering these receptors in inactive pools via Axin binding (Haÿ et al., 2009; Revollo et al., 2019). In this study, we found that knockdown of N-cadherin promoted the β-catenin nucleus translocation and accumulation, while inhibition of β-catenin by XAV939 impeded the odontogenic differentiation of DPSCs. This could be further supported by previous research reporting that the expression of β-catenin was significantly upregulated during odontogenic differentiation of DPSCs, and knockdown of β-catenin disrupted odontogenic differentiation, which could be reversed by the lithium chloride-induced accumulation of β-catenin (Han et al., 2014). Besides, mounting evidence shows that numerous regulators, including but not limited to enhancer of zeste homolog 2, Stathmin, vacuolar protein sorting 4B, R-Spondin 2, and lncRNA *DANCR*, regulate the odontogenic differentiation of DPSCs through the Wnt/β-catenin pathway (Chen et al., 2016; Li et al., 2018; Pan et al., 2019; Zhang et al., 2019; Gong et al., 2020).

Moreover, we found that knockdown of N-cadherin increased the expression of odontogenic markers Runx2 and DSPP. Studies have illustrated that β-catenin binds to the Runx2 promoter and controls its transcription (Gaur et al., 2005), and odontogenic differentiation increases such binding (Han et al., 2014). Runx2 upregulates DSPP expression through binding to the 5′-TACCTCA (−3950 to −3944 bp) and 5′-ACCACA (−3106 to −3101 bp) specific sites in the *DSPP* promoter (Guo et al., 2019). Hence, we propose that N-cadherin silencing induced β-catenin nucleus translocation and accumulation, which then promoted β-catenin binding to the Runx2 promoter and the expression of Runx2 and DSPP, thus stimulating odontogenic differentiation of DPSCs. However, it has been reported that Runx2 needs to be down-regulated to acquire full odontoblast differentiation for dentinogenesis (Miyazaki et al., 2008). The stage-specific roles of Runx2 in odontoblast differentiation and the underlying mechanism warrants further exploration in future studies.

Pulp regeneration, in its most strict sense, must include functional re-innervation of the pulp–dentin complex (Diogenes, 2020). Interestingly, N-cadherin has been implicated in regulating the differentiation of neural progenitor cells during development (Miyamoto et al., 2015), as well as the neurogenic differentiation of menstrual blood-derived endometrial stem cells (Liu et al., 2018). Since DPSCs originate from neural crest cells and are able to differentiate into neural-like cells (Gronthos et al., 2002; Rafiee et al., 2020), how N-cadherin regulates the neurogenic differentiation of DPSCs warrants further investigation.

## Conclusion

**FIGURE 7:**
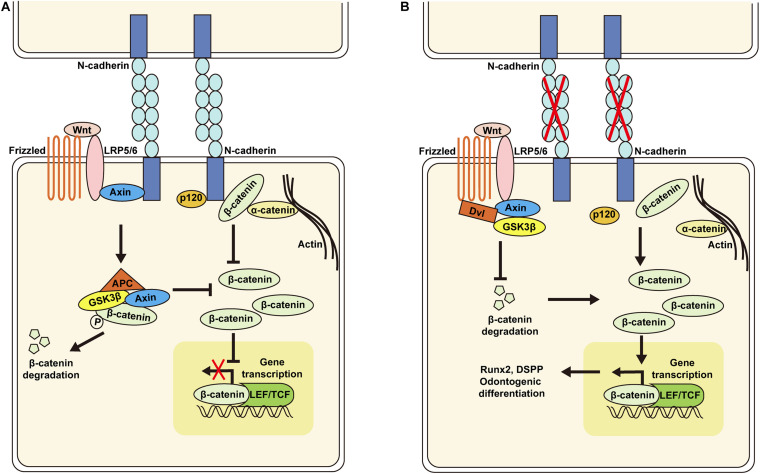
Schematic of the proposed model for N-cadherin/β-catenin signaling in the regulation of odontogenic differentiation of dental pulp stem cells (DPSCs). N-cadherin can sequester β-catenin at the cell–cell adhesion site and associate with the Wnt co-receptor LRP5/6 and Axin to inhibit β-catenin signaling **(A)**. N-cadherin downregulation can promote β-catenin release at the cell membrane and decrease interaction with LRP5/6, resulting in β-catenin signaling activation, Runx2 and DSPP upregulation, and odontogenic differentiation of DPSCs **(B)**.

The results presented here demonstrate that knockdown of N-cadherin enhances β-catenin activity possibly through release of β-catenin at the cell membrane and decreased interaction with the Wnt coreceptor LRP5/6, resulting in Runx2 and DSPP upregulation, and odontogenic differentiation of DPSCs (Figure 7). These data may help to guide DPSC behavior by tuning the N-cadherin-mediated cell–cell interactions, with implications for pulp regeneration.

## Data Availability Statement

The original contributions presented in the study are included in the article/supplementary material, further inquiries can be directed to the corresponding author/s.

## Ethics Statement

The studies involving human participants were reviewed and approved by the Ethics Committee of Nanfang Hospital, Southern Medical University, Guangzhou, China. The patients/participants provided their written informed consent to participate in this study. The animal study was reviewed and approved by the Ethics Committee of Nanfang Hospital, Southern Medical University, Guangzhou, China.

## Author Contributions

ZD, WY performed the experiments, analyzed the data, and wrote the manuscript. XD, MC, and QQ participated in the experiments and revised the manuscript. WZ and BW designed the study and revised the manuscript. All authors have read and approved the final version of the manuscript.

## Conflict of Interest

The authors declare that the research was conducted in the absence of any commercial or financial relationships that could be construed as a potential conflict of interest.
